# The diagnostic value of salivary cortisol and salivary cortisone in patients with suspected hypercortisolism

**DOI:** 10.3389/fendo.2022.1028804

**Published:** 2022-11-23

**Authors:** Vendela Berndt, Per Dahlqvist, Jennie de Verdier, Henrik Ryberg, Oskar Ragnarsson

**Affiliations:** ^1^ Department of Endocrinology, Sahlgrenska University Hospital, Göteborg, Sweden; ^2^ Department of Internal Medicine and Clinical Nutrition, Institute of Medicine, Sahlgrenska Academy, University of Gothenburg, Göteborg, Sweden; ^3^ Department of Public Health and Clinical Medicine, Umeå University, Umeå, Sweden; ^4^ Department of Clinical Chemistry, Sahlgrenska University Hospital, Göteborg, Sweden

**Keywords:** adrenal incidentaloma, mild autonomous hypercortisolism, Cushing´s syndrome, salivary cortisol, salivary cortisone

## Abstract

**Background:**

Diagnosing endogenous hypercortisolism remains a challenge, partly due to a lack of biochemical tests with good diagnostic accuracy.

**Objectives:**

To evaluate the diagnostic value of salivary cortisol and cortisone in patients with suspected hypercortisolism.

**Methods:**

Retrospective study including 155 patients with adrenal incidentaloma, and 54 patients with suspected Cushing´s syndrome (CS). Salivary samples were collected at home, at 11 p.m., and at 8 a.m. following an over-night dexamethasone suppression test (DST). Salivary cortisol and cortisone were measured with liquid chromatography-tandem mass spectrometry.

**Results:**

Ten of 155 patients with adrenal incidentaloma were considered to have autonomous cortisol secretion (ACS). Using previously established cut-offs, all patients with ACS had elevated plasma-cortisol (>50 nmol/L) following DST, 9/10 had elevated late-night salivary cortisone (>15 nmol/L) whereas only 4/10 had elevated late-night salivary cortisol (LNSC; >3 nmol/L) compared to 35%, 9% and 8%, respectively, of the 145 patients with non-functioning adrenal incidentaloma. Six (60%) patents with ACS had elevated salivary cortisol and cortisone at 8 a.m. following DST compared to 9% and 8%, respectively, of patients with non-functioning adrenal incidentaloma. One of 6 patients with overt CS had a normal LNSC and one had normal late-night salivary cortisone, while all had increased salivary cortisol and cortisone following DST.

**Conclusion:**

LNSC is not sufficiently sensitive or specific to be used for screening patients with suspected hypercortisolism. Instead, late-night salivary cortisone seems to be a promising alternative in patients with adrenal incidentaloma and salivary cortisone at 8 a.m. following DST in patients with suspected CS. Larger studies are needed to confirm these findings.

## Introduction

Diagnosing Cushing’s syndrome (CS) and autonomous cortisol secretion (ACS) in patients with adrenal incidentaloma remains a challenge, partly due to a lack of biochemical tests with high diagnostic accuracy (1, 2). Upon clinical suspicion of CS, current guidelines recommend biochemical screening with either a 1 mg overnight dexamethasone suppression test (DST), 24-hour urinary free-cortisol or late-night salivary cortisol (LNSC) (2). For patients with adrenal incidentaloma, DST is the most commonly recommended first-line screening test for ACS (1–5). However, the low specificity of DST for diagnosing CS and ACS is problematic (6).

LNSC is well-established as a first-line screening test for patients with CS (7–10). The diagnostic value of LNSC for detecting ACS in patients with adrenal incidentaloma is, however, less well established (6). The limited number of studies published to date have shown that LNSC has a relatively low sensitivity for predicting ACS, and subsequently conclude that the test is not suitable for screening of this disorder (11–16).

We have recently demonstrated that salivary cortisone at 11 p.m., as well as salivary cortisol and cortisone at 8 a.m. following 1-mg overnight DST, all have high diagnostic accuracy for CS (17). Also, in a recent study from Norway, salivary cortisone at 8 a.m. following 1-mg overnight DST was found to be useful in patients with hypercortisolism, both CS and ACS (18).

The aim of this study was to evaluate the diagnostic value of LNSC, and late-night salivary cortisone, as well as salivary cortisol and salivary cortisone following DST, both in patients with adrenal incidentaloma and in patients with suspected CS.

## Methods

### Study design

This was a retrospective study performed at the Department of Endocrinology at the Sahlgrenska University hospital. All patients who provided at least one saliva sample for measurement of cortisol and cortisone between April 2018 and November 2020 were identified by a search in an administrative program at the Department of Clinical Chemistry at the hospital. Medical records were reviewed to gather information on clinical characteristics, including information on factors known to affect cortisol concentrations, the indication for testing, and biochemical test results.

### Patients

pt?>In total, 398 sets of saliva samples from to 319 patients were analyzed during the study period. In patients that collected more than one set of salivary samples, the first set of samples was used in the final analysis. However, if the earliest set of samples was incomplete, the first complete set was included instead.

**Figure 1:**
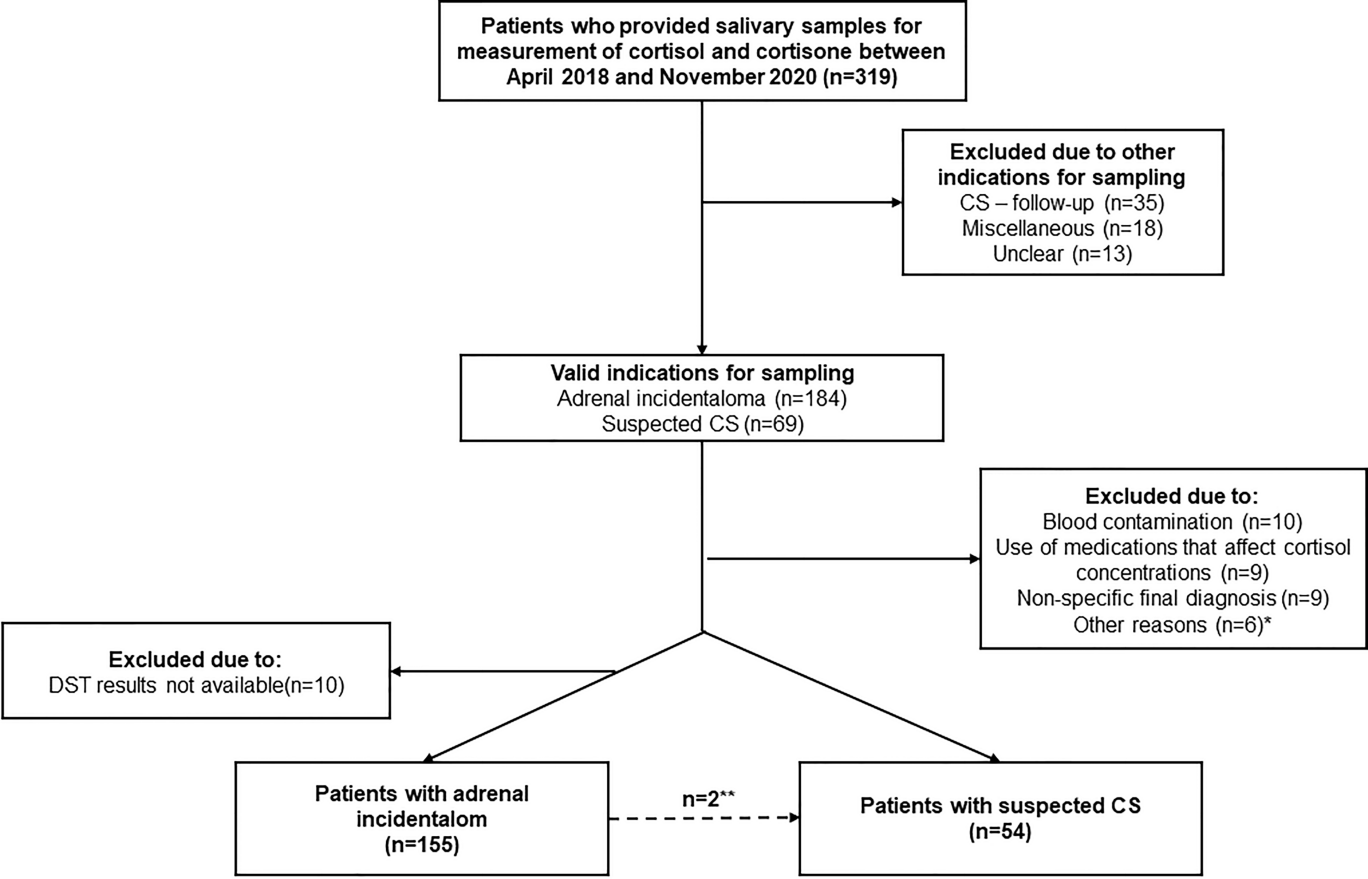
Summary of the study population, 319 patients who provided salivary samples for measurement of cortisol and cortisone between April 2018 and November 2020. *Including one patient with pheochromocytoma, one with primary aldosteronism, one with iatrogenic CS, **Two patients with adrenal incidentaloma had clinically overt CS and were included in the analysis of patients with suspected CS.

The indication for sampling was adrenal incidentaloma in 184 (58%) patients and suspected CS in 69 (22%) (**Figure 1**). Patients with other or unknown indication for sampling were excluded [n=66 (21%)]. Of 253 patients with either adrenal incidentaloma or suspected CS, 10 were excluded due to suspected contamination of the saliva samples (see below), nine due to use of medications known to affect cortisol concentrations, and 15 due to other reasons (**Figure 1**). Furthermore, 10 patients with adrenal incidentaloma were also excluded due to unavailable results from DST. Thus, 209 patients were included for the final analysis (**Figure 1**).

Currently, there is no consensus regarding diagnostic criteria for ACS (6). Therefore, in this study, no predefined diagnostic criteria were used. Instead, the diagnosis was provided through a real-life clinical assessment made by the treating endocrinologist. In general, ACS was diagnosed in patients with adrenal incidentalomas, without overt clinical signs of CS, who had at least two positive diagnostic tests (DST, LNSC, urinary free cortisol and/or plasma-ACTH) indicating abnormal HPA-axis function. Similarly, in patients considered to have non-functioning adrenal incidentaloma, ACS was ruled out based on clinical evaluation showing absence of hypercortisolism-related clinical features and comorbidities, and/or normal biochemical testing (DST, LNSC, urinary free cortisol and/or plasma-ACTH).

The diagnosis of CS was based on clinical symptoms compatible with the syndrome, in combination with at least two biochemical screening tests showing hypercortisolism (2).

### Collection of salivary samples

The saliva samples were collected at home using Salivette^®^ Cortisol tubes (Sarstedt, Nümbrecht, Germany) after oral and written instructions on how to properly collect the saliva had been provided (17). The patients were instructed to collect the first salivary sample between 10 and 11 p.m. by chewing on the foam stick until it was completely saturated with saliva. Thereafter, the patients ingested 1 mg dexamethasone. The following day, the second salivary sample was collected between 6 and 8 a.m. The patients were instructed to avoid intense physical exercise during the day of saliva collection, to avoid smoking and use chewing tobacco, eat, or brush their teeth for one hour before saliva collection. Drinking fluids was allowed 30 minutes before the sampling. Minutes before the sampling, the patients were instructed to rinse their mouths with water.

### Analytical methods and reference ranges

The cortisol and cortisone concentrations in saliva were measured by liquid chromatography-tandem mass spectrometry (LC-MS/MS). The system used was an Acquity UPLC with a Xevo TQS, equipped with a BEH C18 1.7 μm 2.1 × 100 mm analytical column, all from Waters. As buffer A, 20% methanol and 0.1% formic acid in water was used, buffer B was 20% acetonitrile in methanol. Prior to the analysis, the samples were extracted by using Methyl *tert*-butyl ether and ISOLUTE SLE+ 200 μL Supported Liquid Extraction plates. The coefficients of variation were 6% for cortisol at concentrations between 2.2 and 18.4 nmol/L and 7% for cortisone at concentrations between 4.7 and 26.8 nmol/L. The same LC-MS/MS assay was used to measure UFC. P-cortisol was measured by using a radioimmunoassay (Roche Cobas, Cortisol-II) with a coefficient of variation of 2-3%.

Based on our previous study (17), the upper limit of normal for salivary cortisol and cortisone concentrations, were as follows:

- LNSC <3 nmol/L
- Sa-cortisone at 11 p.m. <15 nmol/L
- Sa-cortisol at 8 a.m. following DST <1 nmol/L
- Sa-cortisone at 8 a.m. following DST <5 nmol/L

In general, p-cortisol following DST <50 nmol/L was considered to exclude hypercortisolism, while values >138 nmol/L were considered to be highly suggestive for hypercortisolism (1, 2).

The upper limit of normal for UFC was 136 nmol/L. In a small minority of patients, another method was used for measuring UFC with a upper limit of normal of 191 nmol/L. The lower limit of normal for p-ACTH was 2.0 pmol/L.

Contamination with exogenous hydrocortisone, or blood, in the salivary samples was determined by calculating the salivary-cortisol:cortisone ratio as previously described (17). Samples with a ratio over the 97.5 percentile for the whole cohort (1.0) were considered to be contaminated.

### Statistics

IBM Statistics SPSS version 26 and R were used for statistical analyses.

For comparison of categorical variables, presented as n (%), chi-square test or the Fisher´s exact test were used. Normally distributed numerical variables are described as mean ± standard deviation and non-normally distributed variables as median (interquartile range). Independent t-test was used for comparison between normally distributed variables and the Mann-Whitney U-test for non-normally distributed variables. The level of statistical significance was set at p <0.05.

Receiver-operating characteristic (ROC) analyses were created for each test and presented as area under the curve (AUC) together with 95% confidence intervals (95% CI). DeLong test was used to compare the AUC between the tests, using DST as a reference.

### Ethics

This study was approved by the ethical committee of the University of Gothenburg, (Dnr 814-18), and conducted according to the Declaration of Helsinki.

## Results

In total, 209 patients were included in the study, 157 patients with adrenal incidentaloma and 52 patients with suspected CS. Two patients with adrenal incidentaloma had clinically overt CS, one finally diagnosed with Cushing’s disease and one with cortisol-producing adrenal adenoma, and were therefore included in the analysis of patients with suspected CS. Thus, in the final analysis, data from 155 patients with adrenal incidentaloma, and 54 patients with suspected CS, were included (**Figure 1**).

### Patients with adrenal incidentalomas

Among the 155 patients with adrenal incidentaloma, 145 (94%) were considered to have non-functioning adrenal adenoma and 10 (6%) to have ACS. Age, gender distribution, BMI, and the prevalence of hypertension, diabetes and tobacco use didn’t differ between these groups (**Table 1**).

**Table 1:** Characteristics of patients with non-functioning adrenal incidentaloma and autonomous cortisol secretion who provided salivary samples for analysis of cortisol and cortisone during the study period (April 2018 – November 2020).

	Non-functioning adrenal adenoma (n=145)	Mild autonomous cortisol secretion (n=10)	*p*		
**Age (years)**	62 ± 12	62 ± 10	1.0		
**Women**	92 (63)	7 (70)	1.0		
**BMI (kg/m^2^)**	28.5 ± 5.8	27.5 ± 5.4	0.6		
**Diabetes**	28 (19)	2 (20)	1.0		
**Hypertension**	67 (47)	6 (60)	0.4		
				**Sensitivity**	**Specificity**
**Post-DST P-cortisol >50 nmol/L**	51 (35)	10 (100)	<0.001	100%	65%
**Post-DST P-cortisol >138 nmol/L**	9 (6)	6 (60)	<0.001	60%	94%
**LNSC >3 nmol/L**	11 (8)^**^	4 (40)	0.009	40%	92%
**Late-night Sa-cortisone >15 nmol/L**	13 (9)^**^	9 (90)	<0.001	90%	91%
**Post-DST Sa-cortisol >1 nmol/L**	12 (9)^***^	6 (60)	<0.001	60%	91%
**Post-DST Sa-cortisone >5 nmol/L**	11 (8)^***^	6 (60)	<0.001	60%	92%

Categorical variables are presented as n (%) and continuous variables as mean ( ± SD). Independent t-test, Chi-square test or Fisher´s exact test were used to analyze statistical differences between the groups. ^**^ Not performed in two patients, ^***^ Not performed in three patients.

BMI, body mass index; DST, 1-mg-dexamethasone suppression test; LNSC, late-night salivary cortisol; P, plasma; Sa, salivary.

Of 145 patients with non-functioning adrenal adenoma, 51 (35%) had P-cortisol >50 nmol/L and 9 (6%) had P-cortisol >138 nmol/L after DST, compared to 10 out of 10 and 6 out of 10 patients with ACS, respectively (**Table 1**). Eleven of 143 (8%) patients with non-functioning adrenal adenoma had LNSC >3 nmol/L and 13 (9%) had late-night Sa-cortisone >15 nmol/L, compared to 4 out of 10 (40%) and 9 out of 10 (90%) patients with ACS, respectively (**Table 1**). Six (60%) patents with ACS had elevated salivary cortisol and cortisone at 8 a.m. following DST compared to 9% and 8%, respectively, of patients with non-functioning adrenal incidentalomas. Thus, in patients with adrenal incidentaloma the best diagnostic performance in terms of high sensitivity (90%) in combination with high specificity (91%) for diagnosing ACS was achieved by using late-night Sa-cortisone (**Table 1**).

**Figure 2:**
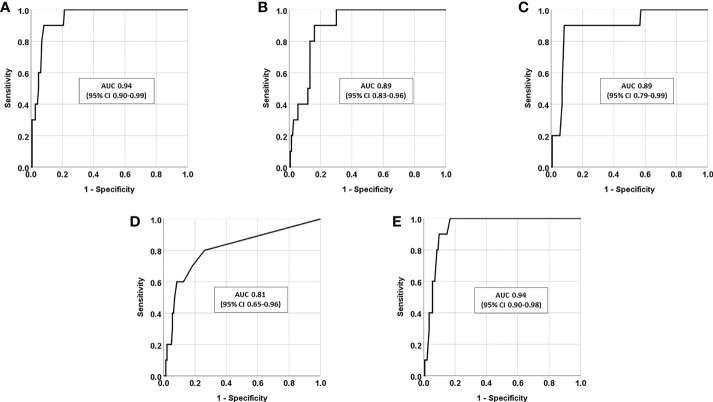
Receiver-operating characteristic (ROC) curves presented as area under the curve (AUC) together with 95% confidence intervals (95% CI), in patients with adrenal incidentaloma (n=155) for **(A)** P-cortisol following dexamethasone suppression test (DST), **(B)** late-night Sa-cortisol (LNSC), **(C)** late-night Sa-cortisone, **(D)** Sa-cortisol at 8 a.m. following DST, and **(E)** Sa-cortisone at 8 a.m. following DST.

In a ROC analysis, the greatest AUC was found for P-cortisol following DST and late-night Sa-cortisone (**Figure 2**). However, no significant differences were found between the AUCs for the salivary tests and the AUC for P-cortisol following DST. P-cortisol following DST at 107 nmol/L gave the best combination of high sensitivity (90%) and specificity (92%).

One patient with ACS (case 1, **Table 2**) was treated surgically with unilateral adrenalectomy and one patient, (case 3, **Table 2**) received pharmacological treatment with Metyrapone. One of these patients had normal LNSC. The remaining 8 patients were treated conservatively.

**Table 2:** Individual data on patients with adrenal incidentalomas diagnosed with autonomous cortisol secretion.

**Case no.**	**Age/Sex**	**BMI**	**Hyper-tension**	**Diabetes**	**UFC**	**P-cortisol after DST**	**P-ACTH**	**LNSC**	**Late-night Sa-cortisone**	**Sa-cortisol after DST**	**Sa-cortisone after DST**
		**Kg/m^2^**			nmol/24-hr^*^	***nmol/L***	pmol/L^*^	nmol/L^*^	nmol/L^*^	nmol/L^*^	nmol/L^*^
1	35/F	25.1	No	No	147	**150 ˄**	**1.0 ˅**	2.4	**16.0 ˄**	**1.6 ˄**	**9.2 ˄**
2	71/F	17.3	No	No	**191 ˄**	**410 ˄**	**0.3 ˅**	**6.8 ˄**	**36.0 ˄**	**5.1 ˄**	**32.0 ˄**
3	69/F	25.2	Yes	No	92	**110 ˄**	**1.8 ˅**	**5.6 ˄**	**16.0 ˄**	**1.0 ˄**	**5.8 ˄**
4	61/F	31.4	Yes	No	67	**160 ˄**	–	1.4	**16.0 ˄**	0.5	4.4
5	67/M	31.2	No	No	**209 ˄**	**66 ˄**	2.4	**4.2 ˄**	**18.0 ˄**	0.6	4.9
6	66/F	33.9	Yes	Yes	78	**270 ˄**	–	**13.0 ˄**	**38.0 ˄**	**1.7 ˄**	**12.0 ˄**
7	63/M	23.5	Yes	No	131	**260 ˄**	–	2.0	**17.0 ˄**	**1.1 ˄**	**11.0 ˄**
8	59/F	25.2	No	Yes	**236 ˄**	**120 ˄**	**1.6 ˅**	2.1	5.5	**3.4 ˄**	3.5
9	59/M	34.9	Yes	No	80	**120 ˄**	2.9	2.3	**16.0 ˄**	0.7	**5.7 ˄**
10	66/F	26.8	Yes	No	20.2	**190 ˄**	**0.9 ˅**	2.2	**18.0 ˄**	0.5	4.9

^*^ Reference ranges: UFC <136 nmol/L, P-ACTH 2-11 pmol/L, LNSC <3 nmol/L, Late-night Sa-cortisone <15 nmol/L, Sa-cortisol after DST <1 nmol/L, Sa-cortisone <5 nmol/L. Abnormal results are marked in bold style and with ˅ (decreased) or ˄ (increased).

ACTH, adrenocorticotropic hormone; BMI, body mass index; DST, 1-mg-dexamethasone suppression test; F, female; LNSC, late-night salivary cortisol; M, male; P, plasma; Sa, salivary; UFC, urinary free cortisol.

In a separate analysis, patients with adrenal incidentaloma were instead categorized according to whether P-cortisol following DST was above or below 138 nmol/L. Among the patients with P-cortisol < 138 nmol/L after DST (n=140), LNSC was >3 nmol/L in 11 (8%), late-night Sa-cortisone >15 nmol/L in 14 (10%), salivary cortisol at 8 a.m. following DST >1 nmol/L in 9 (6%) and salivary cortisone at 8 a.m. following DST >3 nmol/L in 32 (23%). In comparison, of patients with P-cortisol >138 nmol/L after DST (n=15), two (13%) had LNSC >3 nmol/L (*P*=0.5 vs patients with P-cortisol < 138 nmol/L after DST), 7 (47%) had late-night Sa-cortisone >15 nmol/L (P<0.001), 6 (40%) had salivary cortisol at 8 a.m. following DST >1 nmol/L (P<0.001) and 10 (67%) had salivary cortisone at 8 a.m. following DST >3 nmol/L (P<0.001).

### Patients with suspected Cushing´s syndrome

In total, 54 patients, 45 women (87%) and 9 men (13%), with suspected CS were included in the analysis. CS was ruled out in 47 (87%) patients and confirmed in 7 (13%) patients, 4 with Cushing’s disease, two with cortisol-producing adrenal adenoma and one with ectopic CS (**Table 3**).

**Table 3:** Characteristics of patients with suspected Cushing’s syndrome.

	CS ruled out (n=47)	CS (n=7)	*p*		
**Age (years)**	40 ± 17	58 ± 8	0.006		
**Women**	40 (85%)	6 (86%)	1.0		
**BMI (kg/m^2^)**	30.7 ± 8.7	28.8 ± 5.2	0.6		
**Diabetes**	8 (17%)	2 (29%)	0.6		
**Hypertension**	15 (32%)	6 (86%)	0.011		
				**Sensitivity**	**Specificity**
**Post-DST P-cortisol >50 nmol/L**	18/40 (45%)	6/6 (100%)	0.014	100%	55%
**Post-DST P-cortisol >138 nmol/L**	4/40 (10%)	5/6 (83%)	0.001	83%	90%
**UFC**	3/28 (11%)	3/6 (50%)	0.053	50%	89%
**LNSC >3 nmol/L**	4/45 (9%)	5/6 (83%)	<0.001	83%	91%
**Late-night Sa-cortisone >15 nmol/L**	3/45 (7%)	5/6 (83%)	<0.001	83%	93%
**Post-DST Sa-cortisol >1 nmol/L**	7/43 (16%)	7/7 (100%)	<0.001	100%	84%
**Post-DST Sa-cortisone >5 nmol/L**	4/43 (9%)	7/7 (100%)	<0.001	100%	91%

Categorical variables are presented as n/total n of patients (%) and continuous variables as mean ( ± SD). Independent t-test, Chi-square test and Fisher´s exact test were used to analyze statistical differences between the groups.

BMI, body mass index; CS, Cushing’s syndrome; DST, 1-mg-dexamethasone suppression test; LNSC, late-night salivary cortisol; P, plasma; Sa, salivary; UFC, urinary free cortisol.

One patient with CS had P-cortisol <138 nmol/L following DST, one had normal LNSC and one had normal late-night salivary cortisone (**Table 4**). Moreover, UFC was normal in three out of six patients (**Table 4**, cases 1-3). Following DST, all seven patients with CS had Sa-cortisol and cortisone above the normal range. The best diagnostic performance in terms of high sensitivity (100%) in combination with high specificity (91%) for diagnosing CS was achieved by using Sa-cortisone following DST (**Table 3**).

**Table 4:** Individual data on patients diagnosed with Cushing’s syndrome.

**Case no.**	**Age/Sex**	**UFC**	**P-cortisol after DST**	**P-cortisol at 11 p.m.**	**P-ACTH**	**LNSC**	**Late-night Sa-cortisone**	**Sa-cortisol after DST**	**Sa-cortisone after DST**	**Final diagnosis**
		nmol/24-hr^*^	***nmol/L***	***nmol/L***	pmol/L^*^	nmol/L^*^	nmol/L^*^	nmol/L^*^	nmol/L^*^	
1	46/F	124	**160 ˄**	**440 ˄**	8.8	**7.0 ˄**	**28.0 ˄**	**1.1 ˄**	**8.7 ˄**	CD
2	57/F	49.6	**170 ˄**	–	**1.6 ˅**	**4.4 ˄**	11.4	**3.2 ˄**	**10.0 ˄**	CPAA
3	69/F	125	–	**250 ˄**	13.0	2.8	**30.0 ˄**	**1.4 ˄**	**18.0 ˄**	CD
4	63/M	–	**830 ˄**	**690 ˄**	**23.0 ˄**	–	–	**27.0 ˄**	**113.0 ˄**	CD
5	62/F	**860 ˄**	**821 ˄**	–	**14.0 ˄**	**27 ˄**	**78 ˄**	**8.4 ˄**	**53.0 ˄**	ECS
6	52/F	**660 ˄**	**430 ˄**	–	**0.2 ˅**	**12.0 ˄**	**56.0 ˄**	**7.2 ˄**	**44.0 ˄**	CPAA
7	58/F	**465 ˄**	**120 ˄**	–	5.9	**21.0 ˄**	**60.0 ˄**	**4.9 ˄**	**27.0 ˄**	CD

^*^ Reference ranges: UFC <136 nmol/L, P-ACTH 2-11 pmol/L, LNSC <3 nmol/L, Late-night Sa-cortisone <15 nmol/L, Sa-cortisol after DST <1 nmol/L, Sa-cortisone <5 nmol/L. Abnormal results are marked in bold style and with ˅ (decreased) or ˄ (increased).

ACTH, adrenocorticotropic hormone; BMI, body mass index; CD, Cushing´s disease; CPAA, Cortisol producing pituitary adenoma; DST, 1-mg-dexamethasone suppression test; ECS, Ectopic Cushing´s syndrome; F, female; LNSC, late-night salivary cortisol; M, male; P, plasma; Sa, salivary; UFC, urinary free cortisol.

**Figure 3:**
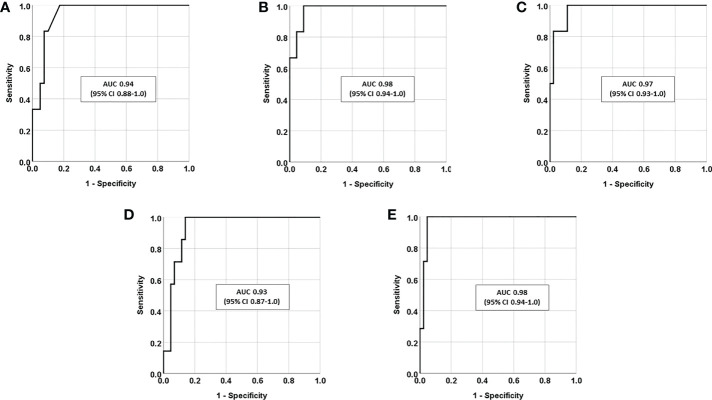
Receiver-operating characteristic (ROC) curves presented as area under the curve (AUC) together with 95% confidence intervals (95% CI), in patients with suspected Cushing’s syndrome (n=54) for **(A)** P-cortisol following dexamethasone suppression test (DST), **(B)** late-night Sa-cortisol (LNSC), **(C)** late-night Sa-cortisone, **(D)** Sa-cortisol at 8 a.m. following DST, and **(E)** Sa-cortisone at 8 a.m. following DST.

The greatest AUC in a ROC analysis was found for LNSC and Sa-cortisone following DST (**Figure 3**), although not significantly different compared to the AUC for P-cortisol following DST.

## Discussion

In this study, we have evaluated the diagnostic value of measuring cortisol and cortisone in saliva in patients with adrenal incidentaloma, and patients with suspected CS. Our findings suggest that LNSC has a low sensitivity for diagnosing ACS in patients with adrenal incidentaloma whereas Sa-cortisone at 11 p.m. had a high sensitivity and specificity for diagnosing the disorder. Furthermore, in patients with suspected CS, Sa-cortisone following DST seem to be a promising diagnostic alternative.

The diagnostic value of measuring LNSC in patients with adrenal incidentaloma has been studied in a limited number of studies (**Table 5**) (11–16). In most of these studies LNSC was analyzed with immunochemical methods, i.e. methods with lower analytical specificity than LC-MS/MS due to a risk of cross-reactions between cortisol and other steroid metabolites (19, 20). To our knowledge, LC-MS/MS has only been used three times before for measuring LNSC in patients with adrenal incidentaloma (12, 15, 16). In one of these studies, the sensitivity of Sa-cortisol <2.8 nmol/L at 11 p.m. was 31%, and the specificity was 83%, for diagnosing ACS in 70 patients with adrenal incidentaloma (12), i.e. in line with the current study. Similar results were found in a study from Norway on 165 patients with adrenal incidentaloma (15), and a study from Italy on 106 patients (16). In fact, due to the low sensitivity, all previous studies, regardless of analytical assay, have concluded that LNSC cannot be recommended as an exclusive screening method for ACS (11–16). Instead, LNSC may be used in combination with other tests to confirm the disorder (11).

**Table 5:** Summary of studies that have evaluated the diagnostic value of salivary cortisol in patients with adrenal incidentaloma.

	Definition of ACS	No of patients	Main findings	Main conclusion	Comments
Masserini, 2009, Milan, Italy	Two of the following: Cortisol after DST >83 nmol/l, 24-h UFC >193 nmol/24 h, and morning ACTH <2.2 pmol/l.	22 ACS of 103 AI (21%)	LNSC 5.1 nmol/l, had 23% sensitivity and 88% specificity for diagnosis of ACS	LNSC is not suitable as a screening test for ACS. May be used with other tests to confirm ACS	LNSC measured with immunofluorimetrical assay
Palmieri, 2013, Milan, Italy	Two of the following: Cortisol after DST >83 nmol/l, 24-h UFC >193 nmol/24 h, and morning ACTH <2.2 pmol/l.	16 ACS of 70 AI (23%)	LNSC >2.8 nmol/l had 31% sensitivity and 83% specificity for predicting ACS	LNSC useful in combination with DST for diagnosing ACS, but not useful as a single criterion	LNSC measured with LC-MS/MS
Ceccado, 2017, Padova, Italy	S-cortisol >138 nmol/L after DST, or s-cortisol after DST 50–138 nmol/L and one of the following: ACTH <10 ng/L, high LNSC, or high UFC	30 ACS of 164 AI (18%)	Median LNSC higher in patients with ACS than non-ACS, but without a reliable cutoff	Consider UFC together with DST to reduce false-positives. LNSC not suitable as a screening test for ACS	Main focus is on UFC, not LNSC LNSC measured with a radio-immunometric assay
Ueland, 2017, Norway	S-cortisol >50 nmol/L after DST, low morning P-ACTH and at least one ACS related comorbidity.	25 ACS of 131 AI	13 of 25 patients with ACS had elevated post-DST Sa-cortisol and 23 had elevated Sa-cortisone	Post-DST Sa-cortisone useful, especially for diagnosing of hypercortisolism	S-dexamethasone and salivary cortisol and cortisone measured with LC-MS/MS. Controls included and used to calculate reference ranges
Ceccato, 2018, Italy	S-cortisol >50 nmol/L after DST	46 ACS of 106 AI	LNSC similar in patients with or without S-cortisol >50 nmol/L after DST	LNSC not useful to discriminate between non-functioning AI and ACS	Salivary cortisol and cortisone measured with LC-MS/MS
Ueland, 2020, Norway	S-cortisol >50 nmol/L after DST	83 ACS of 165 AI	LNSC false-positive in 23/63 and false-negative in 38/69	LNSC not useful to discriminate between non-functioning AI and ACS	S-dexamethasone and salivary cortisol and cortisone measured with LC-MS/MS. Patients with low dexamethasone bioavailability and patients using oral estrogens excluded
Araujo-Castro, 2021, Spain	S-cortisol after DST >138 nmol/L	19 ACS of 197 AI (10%)	LNSC >157 nmol/l had 88% specificity and 47% sensitivity for identifying ACS. 25% sensitivity for S-cortisol > 50 nmol/L	LNSC has a low reliability for diagnosing ACS	LNSC measured with Electroimmuno-chemiluminescence assay

ACS, Autonomous cortisol secretion; ACTH, adrenocorticotropic hormone; AI, Adrenal incidentaloma; DST, 1-mg over-night dexamethasone suppression test; LC-MS/MS, liquid chromatography tandem mass spectrometry; LNSC, late-night salivary cortisol; P, plasma; S, serum; Sa, salivary; UFC, urinary free cortisol.

Bäcklund et al. recently found that Sa-cortisone at 11 p.m. had a higher sensitivity than LNSC for diagnosing CS (17). Our study, showing that 9 of 10 patients with ACS had elevated Sa-cortisone at 11 p.m., suggest that Sa-cortisone may be of greater diagnostic value than LNSC also in patients with adrenal incidentaloma. Similarly, simultaneous measurement of LNSC and Sa-cortisone was recently recommended by Mohamed and colleagues (8). In addition, measuring Sa-cortisol and Sa-cortisone simultaneously enables detection of contamination by exogenous hydrocortisone in the samples. Further studies investigating the value of Sa-cortisone as a part of the diagnostic work-up for adrenal incidentaloma are needed, including efforts to identify biomarkers of clinically relevant ACS that warrants adrenalectomy.

Sa-cortisol and Sa-cortisone were inadequately suppressed following DST in 6 of 10 patients with ACS, whereas inadequate suppression of Sa-cortisol and Sa-cortisone was found only in 9 and 8%, respectively, of patients with non-functioning adrenal incidentalomas, giving a specificity of 91/92%. Sa-cortisol and Sa-cortisone at 8 a.m. following DST has to our knowledge only been studied once before in the context of screening for ACS (18). In that study, 13 of 25 patients with ACS had elevated post-DST Sa-cortisol and 23 of 25 had elevated Sa-cortisone, with 90% and 82% specificity, respectively. Analysis of Sa-cortisol and Sa-cortisone following DST has also been used in two previous studies to screen patients with suspected CS, both showing a high sensitivity (95-100%) and specificity (80-95%) (17, 18), in agreement with our findings. Thus, Sa-cortisol and/or Sa-cortisone following DST are promising diagnostic tools in patients with adrenal incidentaloma as well as patients with suspected CS.

Currently, there is no international consensus on how ACS in patients with adrenal incidentalomas should be diagnosed, and different recommendations have been provided (1–5, 21–23). However, due to the high sensitivity, DST is the most frequently recommended first-line screening method. Indeed, all patients in our study diagnosed with ACS had P-cortisol >50 nmol/L on the DST, supporting a high negative predictive value of P-cortisol <50 nmol/L. The downside of this test is, however, the low specificity. In our study, 35% of patients with non-functioning adrenal incidentaloma had P-cortisol >50 nmol/L following DST, i.e., a “false positive test”, in agreement with previous rapports (6). P-cortisol >138 nmol/L following DST has been suggested for diagnosis of ACS by the National Institute of Health (21). In our study, 10% of all patients with adrenal incidentalomas had P-cortisol >138 nmol/L, providing a more reasonable prevalence of ACS in comparison to a cut-off of 50 nmol/L. However, 40% of the patients diagnosed with ACS in our cohort had P-cortisol <138 nmol/L following DST, illustrating a relatively poor ability to confirm the diagnosis by using this cut-off. Our results, therefore, indicate that DST should not be the only biochemical test used to diagnose mild autonomous hypercortisolism, regardless of the cut-off value used.

In a recent meta-analysis including 139 studies, the accuracy of laboratory tests for CS was investigated (24). The sensitivity was high for all tests, lowest for UFC (94%) and highest for DST (99%). In the current study, one patient with overt CS had normal LNSC, another had P-cortisol <138 nmol/L following DST and half of the patients had normal UFC. Interestingly, however, all patients with overt CS had elevated Sa-cortisol as well as Sa-cortisone at 8 a.m. following DST. Also, in agreement with Bäcklund et al. (17), LNSC and Sa-cortisone at 11 p.m. had a slightly lower sensitivity than salivary samples collected after DST.

The limitations of this study include the limited number of patients, especially the group of patients finally diagnosed with CS, and that two or more saliva samples were not consequently sampled in all patients with suspected CS, emphasizing the need for caution when interpreting the results. Also, due to the retrospective design, the diagnostic criteria for ACS was not predefined. Instead, the diagnosis was based on clinical findings and results from the different cortisol measurement, i.e., a real-life assessment made by several physicians responsible for each patient. Due to the lack of predefined specific criteria, the risk of diagnostic inconsistency cannot be ruled out. Moreover, since the treating clinicians were not blinded to the results of the salivary samples when diagnosing ACS and CS, it is difficult to evaluate independently the diagnostic value of each biochemical test, including salivary cortisol and cortisone. In addition, elevated P-cortisol following DST is often required for diagnosing ACS, which inevitably may have contributed to the high sensitivity of this test. Finally, P-dexamethasone, analysed together with P-cortisol in association with DST, in order to decrease the number of false positive results (18), was not measured in our study. The study has also several strengths, including that all patients who provided salivary samples followed a standardized protocol for collection and a relatively large number of patients with adrenal incidentalomas were included. Furthermore, both salivary cortisol and cortisone were measured, enabling calculation of the cortisol:cortisone ratio and evaluation if the samples were contaminated with exogenous cortisol (hydrocortisone). Another strength is that the salivary samples were analyzed using LC-MS/MS, which is more accurate than other analytical methods used to analyze steroids.

In conclusion, LNSC does not by itself seem to be sufficiently sensitive to screen patients with suspected hypercortisolism, neither in patients with adrenal incidentalomas, nor in patients with suspected CS. However, Sa-cortisone at 11 p.m. and salivary cortisol and cortisone following DST seem to be promising tests in the same context. Nevertheless, further studies on the diagnostic value of salivary cortisol and cortisone are needed. Ideally, this would be done prospectively with a sufficiently large number of patients, where the criteria for ACS would be predefined, and the investigators would be blinded to the results on salivary cortisol/cortisone when the decision on diagnosis is made.

## Data availability statement

The raw data supporting the conclusions of this article will be made available by the authors, without undue reservation.

## Ethics statement

This study was approved by the ethical committee of the University of Gothenburg, (Dnr 814-18). Written informed consent for participation was not required for this study in accordance with the national legislation and the institutional requirements.

## Author contributions

VB: data curation, formal analysis, investigation, methodology, project administration, software, visualisation and writing - original draft preparation. PD, JV, and HR: writing - reviewing and editing. OR: conceptualisation, formal analysis, funding acquisition, methodology, resources, software, supervision, validation, visualisation, and writing - reviewing and editing. All authors contributed to the article and approved the submitted version.

## Acknowledgments

We would like to thank Göran Oleröd and Sara Nadi at the Department of Clinical Chemistry at Sahlgrenska University Hospital, for identifying the eligible patients and providing their salivary test results and Nils Bäcklund for statistical help comparing the ROC curve AUCs.

## Conflict of interest

The authors declare that the research was conducted in the absence of any commercial or financial relationships that could be construed as a potential conflict of interest.

## Publisher’s note

All claims expressed in this article are solely those of the authors and do not necessarily represent those of their affiliated organizations, or those of the publisher, the editors and the reviewers. Any product that may be evaluated in this article, or claim that may be made by its manufacturer, is not guaranteed or endorsed by the publisher.
